# Untangling the role of social support in type 2 diabetes: insights from a mixed methods study in Quito, Ecuador

**DOI:** 10.3389/fpubh.2025.1668181

**Published:** 2025-11-06

**Authors:** Marta Puig-García, Ana Henriques, Andrea Bravo-Díaz, María José Sanchís-Ramón, Mariana Amorim, Sergio Morales-Garzón, Jessica Pinto-Delgado, Elisa Chilet-Rosell, Lucy Anne Parker

**Affiliations:** 1 Department of Public Health, History of Science and Gynaecology, Universidad Miguel Hernández de Elche, Alicante, Spain; 2 CIBER de Epidemiología y Salud Pública (CIBERESP), Madrid, Spain; 3 EPIUnit ITR, Instituto de Saúde Pública, Universidade do Porto, Porto, Portugal; 4 Grupo de Inequidades y Determinantes Ecológicos y Ambientales en Salud (IDEAS), Instituto de Salud Pública, Facultad de Medicina, Pontificia Universidad Católica del Ecuador, Quito, Ecuador

**Keywords:** type 2 diabetes, social support, glycemic control, qualitative research, chronic disease, Ecuador

## Abstract

**Introduction:**

Type 2 diabetes (T2DM) requires sustained behavioral changes to achieve glycemic control (GC) and prevent complications. Social support (SS) is key for self-care in chronic conditions, yet its impact on GC in low-resource settings remains understudied. This study explores the role of SS in T2DM management in a low-income community in Quito, Ecuador.

**Methods:**

We employed a mixed-methods approach with a parallel convergent design. A retrospective cohort study was conducted with 332 adults aged 25–88 years with T2DM, recruited from public health facilities in district D1706. Quantitative data were collected using structured questionnaires, including the multidimensional scale of perceived social support (MSPSS); GC was assessed through clinical records. Logistic regression models were used to examine associations between SS and GC, reporting crude and adjusted odds ratios (ORs) with 95% confidence intervals. In parallel, 16 semi-structured interviews were conducted with adults with T2DM and analyzed thematically using framework analysis.

**Results:**

Of the 332 participants, 263 had at least one glucose measurement and 28.6% achieved good GC. Those with better control reported slightly higher SS. While the overall SS score was not strongly associated with GC, the subscale of family support showed a positive association (adjusted OR: 1.07; 95% CI: 1.00–1.14 per MSPSS point). Qualitative findings highlighted the importance of emotional and instrumental family support, the perceived loss of friendship-based support, and the supportive role of community-based diabetes clubs.

**Discussion:**

The association between family support and glycemic control highlights the need to incorporate family dynamics into T2DM management strategies. However, expanding support networks beyond the family, such as through peer and community-based resources, may further enhance coping capacity and overall wellbeing. Interventions that integrate emotional, instrumental, and informational support from diverse sources could play a critical role in improving self-management and optimizing health outcomes.

## 1 Introduction

Type 2 diabetes mellitus (T2DM) is a major global health challenge, currently affecting over 536 million people worldwide, with numbers projected to rise further, particularly in low- and middle-income settings (1). In Ecuador, the International Diabetes Federation estimated the prevalence of T2DM at 4.7% in 2021 (1). However, local studies reveal substantial regional and population-based variability, with prevalence ranging from 2% to over 10% (2–5). This heterogeneity reflects differences in demographic profiles, socioeconomic disparities, health behaviors, dietary patterns, and unequal access to preventive and healthcare services across urban, rural, and coastal-highland areas. National mortality statistics highlight the disease’s significant burden, ranking diabetes as the second leading cause of death among women and fifth among men (6).

Effective management of this chronic condition requires both medical management by healthcare providers and active self-management by patients (7–9). This includes following key behavioral modifications, such as a balanced diet, regular physical activity and medication adherence. While these recommendations are essential for enhancing glycemic control (GC), preventing complications, and improving quality of life, they often overlook the structural inequalities that make self-management challenging. Individuals from socioeconomically disadvantaged backgrounds face additional barriers such as limited access to healthy food, safe spaces for exercise, and affordable healthcare, which exacerbate their vulnerability to T2DM (10–12). Consequently, interventions promoting self-management without addressing broader social determinants may benefit some individuals, but often fail to reach those most in need, thereby inadvertently reinforcing existing health inequities (13).

Within this context, creating supportive environments is crucial for promoting self-management and managing chronic conditions. Social support (SS) is increasingly recognized as a key factor in sustaining self-care behaviors, enhancing coping strategies, and improving disease awareness among individuals with T2DM (9, 14). Conceptually, SS is a multidimensional construct that encompasses emotional, informational, companionship, and practical support provided by family, friends, peers, and community networks (8, 15). Emotional support includes empathy, understanding, and reassurance, whereas informational support provides guidance, advice, and health-related knowledge. Practical support may involve tangible assistance, including help with daily tasks, financial aid, or ensuring medication adherence. Companionship, on the other hand, fosters a sense of belonging and encourages engagement in social activities (8). Together, these dimensions create an enabling environment for healthier behaviors and improved disease management.

SS operates through various mechanisms such as buffering the negative effects of stress associated with chronic illnesses, enhancing self-efficacy, and facilitating access to resources (16, 17). Importantly, the perception of SS can be positive or negative, and should be differentiated from the objective social network, namely the size and extension of relationships (18, 19). Moreover, the quality and type of support received varies significantly across demographic and cultural backgrounds (8, 17).

Despite growing recognition of its importance, research on the role of SS in T2DM management remains limited in low-income settings, where barriers to effective care are particularly pronounced (10, 20). Addressing this gap in these contexts is crucial for designing interventions that effectively reduce the burden of T2DM and improve health outcomes. Therefore, this study aims to explore the role of SS in T2DM management in a low-income community in Quito, Ecuador, by addressing two questions: (1) What is the association between perceived SS and GC among people living with T2DM? and (2) How do individuals perceive different types and sources of SS in their everyday management of the condition?

## 2 Methods

### 2.1 Study design

The present study is part of the CEAD project—Contextualizing Evidence for Action on Diabetes in low-resource Settings: a Mixed-methods case study in Quito and Esmeraldas, Ecuador. This project was conducted between 2019 and 2024 and used a mixed-methods approach that integrates epidemiological analysis, qualitative and participatory action research in order to examine how global recommendations can be tailored to specific, evidence-based measures to prevent diabetes in local contexts.

A core pillar of the CEAD project is health systems strengthening. In order to evaluate implementation of comprehensive diabetes care, we sought to conduct a retrospective cohort study of individuals living with diabetes in two health districts, and carried out qualitative research with health professionals, patients and families (21).

The present study is based on quantitative data from a subset of 332 individuals from the retrospective cohort in southern Quito and qualitative data from a purposive sample of 16 individuals who lived with T2DM in the area. This mixed methods study employed a parallel convergent design to explore the role of SS in T2DM management and GC (22, 23). Quantitative and qualitative data were first analyzed separately and then compared through triangulation to identify convergent and divergent findings. Integration also occurred iteratively during analysis and interpretation: quantitative results on social support and glycemic control guided exploration of patterns in qualitative interviews, while qualitative findings provided contextual understanding and explanations for observed associations.

### 2.2 Setting

The research reported in this paper was conducted in the district 17D06, an urban health district with 507,499 residents (in 2017) in the south of Quito. This area is characterized by high population density, internal migration, and significant unmet basic needs.

Ecuador’s public health system is structured to provide universal coverage through two main sectors. The Ministry of Public Health (MSP) serves the general population, focusing on disadvantaged groups, while the Social Security Institute (IESS) covers formally employed workers via employer-employee contributions, with additional schemes for rural workers, the military, and the police (24). Primary care is organized into three levels of health care attention. Type A centers serve 2,000–10,000 people, offering basic family medicine, prevention, and health promotion. Type B centers serve 10,000–25,000 people and add specialized services such as nutrition, mental health, and social work. Type C centers serve 25,000–50,000 people, providing both general and specialized care, including gynecology and pediatrics. In health district 17D06, the MSP operates one type C health centre, two Type B facilities, and 25 Type A facilities, while the IESS manages three centers and one hospital.

Diabetes care is included under universal coverage, yet significant barriers persist (25). Institutional fragmentation, weak coordination between primary and specialized care, shortages of trained personnel and diagnostic equipment, and inequitable distribution of services limit effectiveness. Rural and marginalized populations face additional challenges due to distance, transportation costs, and socioeconomic constraints. These factors contribute to delayed diagnoses, inconsistent follow-up, and poor glycemic control among people with T2DM (25).

### 2.3 Quantitative study: retrospective cohort study

#### 2.3.1 Participants

Participants were recruited from a sample of MSP health facilities. Initially, we selected the only type C health centre, and a random selection of six type A/B centers from the 27 available. We aimed to recruit 160 participants from the type C and 40 per A/B center. A randomized list of additional health facilities was generated to serve as replacements until the required sample size was achieved. Facilities were contacted through official channels, and physicians provided patient lists. Initial telephone recruitment was hindered by contact errors, prompting a shift to a mixed approach, combining face-to-face recruitment and scheduled meetings. Consecutive sampling was then applied until the required sample size was achieved.

**Figure 1:**
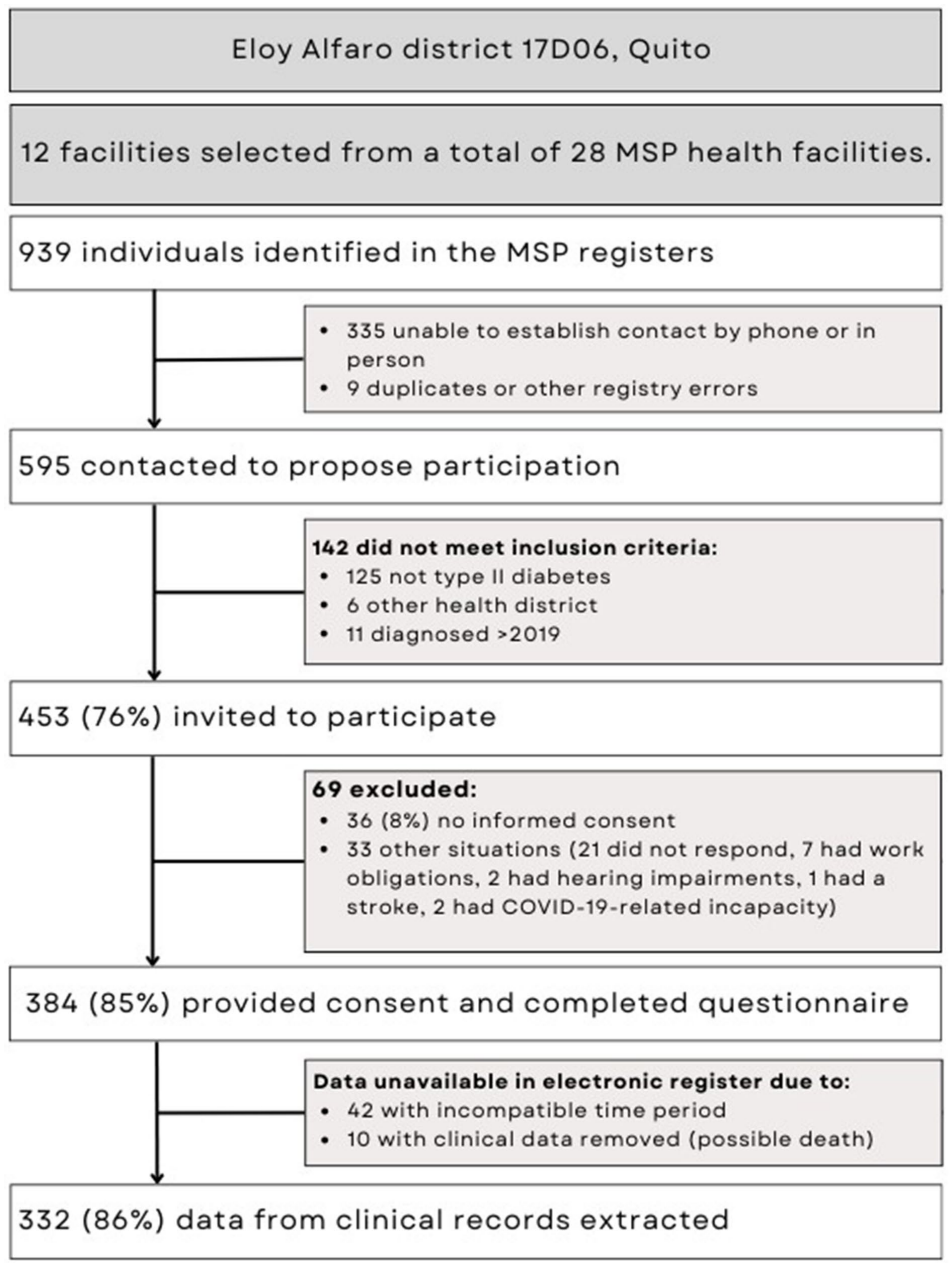
Flowchart of participants with type 2 diabetes included in the quantitative analysis of a mixed-methods study in southern Quito, Ecuador.

Of 939 possible participants who were treated in 12 MSP health centers, we finally included 332 participants, from whom we were able to extract clinical information (Figure 1). Of these, 263 (79%) had at least one record of glucose measurement.

#### 2.3.2 Data collection

The research team, composed of two women and two men, recruited participants between April 2022 and March 2023. The survey collected information about sociodemographic characteristics, access to health services, clinical data, behavioral risk factors, perceived SS and the Diabetes Health Profile-18 (DHP-18). Following informed consent, clinical information regarding treatment, complications, healthcare visits, health metrics (weight, BMI, blood pressure), and glucose status was retrieved from participants’ digital medical records. The follow-up period covered the 12 months following each participant’s survey date. Clinical data extraction was conducted between July and September 2024.

#### 2.3.3 Variables

The main outcome variable, controlled glycaemia, was extracted from clinical records prioritizing measures hierarchically based on clinical evidence, using HbA1c, then fasting blood glucose, and finally random blood glucose. When multiple measurements were available, the measurement closest to the one-year follow-up first was prioritized. GC was defined according to ADA guidelines in controlled when HbA1c ≤ 7%, fasting capillary blood glucose between 80 and 130 mg/dL or random blood glucose <180 mg/dL, and in poorly controlled for any other case. As such, individuals who had no glycemic data in their clinical files were included the analysis, classified as “uncontrolled” together with those with poor glycemic results. Sensitive analyses indicated that this categorization did not affect the overall findings (Supplementary Table A). This approach allowed retention of all participants while acknowledging that missing data may introduce some misclassification bias.

We assessed participants’ perceived SS using the multidimensional scale of perceived social support (MSPSS) instrument (26). For this study, we employed the version adapted by Arechabala and Miranda (27), originally developed for Chilean older adults with hypertension and later applied to Chilean patients with T2DM (28). This adaptation uses a 4-point Likert-type scale ranging from 1 (almost never) to 4 (always or almost always), which provides an overall score ranging from 12 to 48, as well as scores for three subscales: family, friends, and significant other, each ranging from 4 to 16. Higher scores indicate a greater perception of SS. The instrument was reviewed to ensure comprehension and cultural relevance in the study context.

Household income was divided into two categories based on the basic salary in Ecuador at the moment of the questionnaire $0–$375 and over $375. The duration of T2DM was calculated as the difference from the self-reported diagnosis date to the interview date and was treated as a continuous variable. We considered oral antidiabetics and insulin treatment for T2DM, and we define polymedication as the use of three or more medications for different chronic indications.

#### 2.3.4 Statistical analysis

We used descriptive statistics to summarize the sociodemographic and clinical characteristics of the study population and compared these characteristics in patients with controlled and uncontrolled glycemic levels. The chi-square test assessed differences in proportions for categorical variables, while the Mann–Whitney U test evaluated median differences for continuous variables. Lastly, we conducted a logistic regression analysis to examine the relationship between the level of SS (overall score and its three subscales) with GC. We calculated both crude and adjusted odds ratios (ORs) along with their corresponding 95% confidence intervals (CIs) for each subscale. We adjusted the ORs for sex, age, and duration of diabetes to account for potential confounding effects. All analyses were conducted using Stata version 15.0 (StataCorp, College Station, TX, USA).

### 2.4 Qualitative study: interviews

#### 2.4.1 Participants and data collection

We conducted in-person interviews with 16 individuals living with T2DM from the district 17D06, Quito in May and June 2023. We used purposive sampling to ensure heterogeneity in terms of gender, age, education level, employment status, ethnic group and socioeconomic status. Sample adequacy was guided by richness and diversity of accounts rather than data saturation (29). We reached out to potential participants by phone explaining the study’s objectives and inviting them to participate on a mutually convenient day and time. Interviews were conducted either at the participant’s residence or within the van used by our research team, based on the participant’s preference. We developed a semi-structured interview script to gather information about participants’ personal experiences managing T2DM, including life changes since diagnosis and resources needed for control (full script in 10.5281/zenodo.15719473). It also explored sources of SS such as family, friends, community, and healthcare providers, and the types of support received (informational, practical, emotional). MP-G conducted the interviews, and MH registered non-verbal information and took notes. The duration of the interviews averaged 36 min, ranging from 14 min to 1 h 10 min. All interviews were audiotaped and transcribed verbatim. Tone of voice, silences and interruptions were registered, contributing to a comprehensive dataset for analysis.

To ensure the trustworthiness of the qualitative component, we followed Guba and Lincoln’s criteria (30, 31). Credibility was strengthened through triangulation between researchers, engagement in the study setting, and consultation with local experts; in addition, member checking was carried out with the community as part of a complementary participatory research. Transferability was supported by detailed descriptions of the study context and participant characteristics. Dependability was ensured through supervision by two researchers not involved in data collection, and confirmability was reinforced by keeping reflexive notes, applying thematic analysis with triangulation during coding and writing, and including participants with diverse characteristics. The semi-structured interview guide was developed based on the study objectives, reviewed by local public health researchers to ensure content validity, and evaluated by a native speaker for linguistic and cultural appropriateness. The guide was iteratively adapted during the first interviews to improve clarity and comprehensiveness.

#### 2.4.2 Framework analysis

The verbatim transcripts were analyzed using the framework analysis (32) in ATLAS.ti 8. MP-G used a line-by-line coding approach to identify relevant pieces of information from the data and conducted inductive coding to capture participants’ perspectives on SS and diabetes management. The initial codes described needs, sources and types of support, as well as attitudes and sociodemographic factors that influenced the previous aspects. After coding the first four transcripts, MP-G refined these codes and grouped them into broader categories, including Types of Support, Sources of Support, Diabetes Management and Attitudes. At this point, AB and MP-G triangulated the data and agreed on the codes and categories used. The refined framework was applied to all transcripts, indexing the data into the agreed-upon categories. Using ATLAS.ti 8, we summarized and presented the data into a framework matrix, structuring the results around sources of support (e.g., family, friends, significant others) as the central theme and explored relationships between SS, diabetes management, and sociodemographic characteristics to develop explanations beyond quantitative findings. We conducted the analysis of the interviews in Spanish, and subsequently, the selected excerpts were translated into English. The original Spanish versions of the excerpts are available in Supplementary material 2.

## 3 Results

The retrospective cohort study sample was predominantly women (81.6%), with a median age of 64 years (IQR: 58–71) (Table 1). Most identified as mestizo (87.9%), with primary school education being the most common level attained (58.1%). More than half lived with a partner (54.1%).

**Table 1:** Sociodemographic and clinical characteristics of the study participants according to the glycemic control (*N* = 332).

Variable	Total *n* (%)	Controlled *n* (%)	Uncontrolled* *n* (%)	*p*-value^a^
Sex				
Men	61 (18.4%)	15 (24.6%)	46 (75.4%)	0.441
Women	271 (81.6%)	80 (29.5%)	191 (70.5%)	
Age, median (IQR) in years	64 (58–71)	63 (55–72)	64 (58–71)	0.468
Ethnicity^b^				
Mestizo	290 (87.9%)	85 (29.3%)	205 (70.7%)	0.344
White	23 (7.0%)	8 (34.8%)	15 (65.2%)	
Afro	5 (1.5%)	0 (0.0%)	5 (100.0%)	
Indigenous	12 (3.6%)	2 (16.7%)	10 (83.3%)	
Education level				
No formal schooling	32 (9.6%)	8 (25.0%)	24 (75.0%)	0.661
Primary school	193 (58.1%)	53 (27.5%)	140 (72.5%)	
Secondary school	78 (23.5%)	23 (29.5%)	55 (70.5%)	
Higher education	29 (8.7%)	11 (37.9%)	18 (62.1%)	
Civil status^b^				
Married	159 (48.0%)	42 (26.4%)	117 (73.6%)	0.679
Separated/divorced	59 (17.8%)	20 (33.9%)	39 (66.1%)	
Single	42 (12.7%)	14 (33.3%)	28 (66.7%)	
Widowed	51 (15.4%)	12 (23.5%)	39 (76.5%)	
Living with partner	20 (6.0%)	6 (30.0%)	14 (70.0%)	
Employment status				
Unpaid/Other	162 (48.8%)	43 (26.5%)	119 (73.5%)	0.506
Self-employed	131 (39.5%)	38 (29.0%)	93 (71.0%)	
Employed	39 (11.7%)	14 (35.9%)	25 (64.1%)	
Household earnings				
<$375	152 (45.8%)	45 (29.6%)	107 (70.4%)	0.577
≥$375	112 (33.7%)	34 (30.4%)	78 (69.6%)	
NS/NC	68 (20.5%)	16 (23.5%)	52 (76.5%)	
Duration of T2DM^b^, median (IQR) in years	12 (6–18) years	10 (6–17) years	13 (6–18) years	0.087
Type of treatment				
No treatment	13 (3.9%)	4 (30.8%)	9 (69.2%)	0.017
Only oral meds	162 (48.8%)	59 (36.4%)	103 (63.6%)	
Only insulin	22 (6.6%)	4 (18.2%)	18 (81.8%)	
Oral + insulin	135 (40.7%)	28 (20.7%)	107 (79.3%)	
Polymedication				
Less than 3 meds	138 (41.6%)	40 (29.0%)	98 (71.0%)	0.900
≥3 meds	194 (58.4%)	55 (28.4%)	139 (71.6%)	
Belongs to health-based support group				
No	226 (68.1%)	67 (29.7%)	159 (70.3%)	0.544
Yes	106 (31.9%)	28 (26.4%)	78 (73.6%)	
Total	332 (100.0%)	95 (28.6%)	237 (71.4%)	

*Uncontrolled: participants who had no glycemic control data recorded during follow-up, as well as those with poor glycemic outcomes.

a *p-*value from Mann–Whitney *U* test for age and duration of T2DM; chi-squared test for the categorical variables.

b Two missing ethnicity; one missing civil status, five missing T2DM duration.

Among individuals with T2DM, GC was achieved in 28.6%, while 50.6% had suboptimal GC, and 20.8% had no glycemic measurements recorded in their clinical files. Glycemic assessment was based on HbA1c in 56.7% of cases, with the rest using capillary glucose (22.1%) or fasting plasma (20.9%), and only one random glucose.

The median duration of T2DM was slightly shorter in the controlled group (10 years, IQR 6–17) compared to the uncontrolled group (13 years, IQR 6–18), (*p* = 0.087). Participants using only oral medications had the highest control rate (36.4%), while those on insulin alone (18.2%) or combined oral and insulin therapy (20.7%) exhibited notably poorer control (*p* = 0.017). The most prevalent comorbidities among participants with T2DM were hypertension (67.5%) and dyslipidemia (43.1%), followed by retinopathy (13.0%) and kidney disease (6.0%). Notably, only one case of amputation was reported (Supplementary Table B).

### 3.1 Relationship between glycemic control and perceived social support

**Figure 2:**
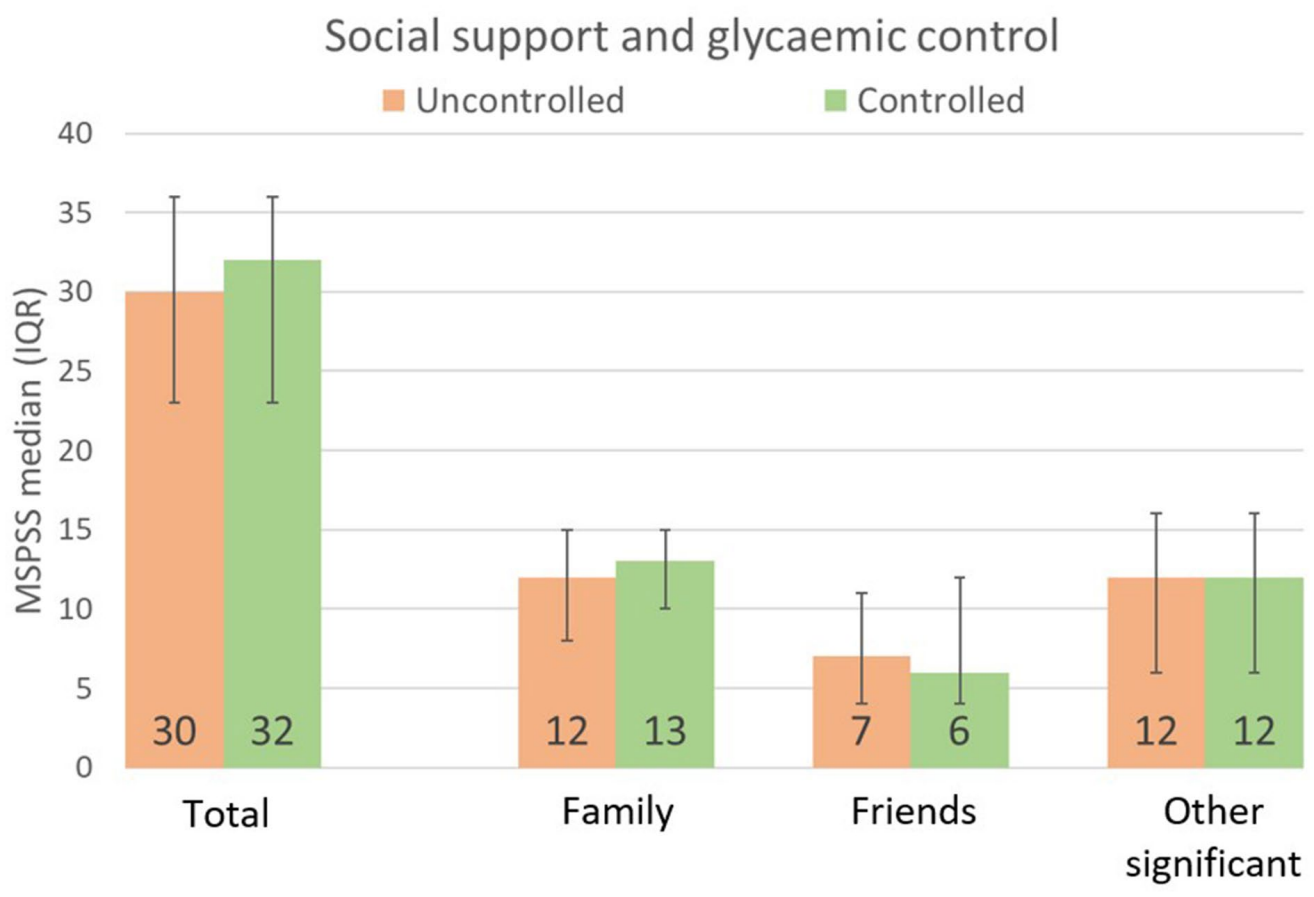
Median multidimensional scale of perceived social support (MSPSS) scores reported by participants with type 2 diabetes according to glycemic control status (*n* = 332).

Participants reported a moderate level of perceived SS (the median MSPSS score was 31 out of a maximum of 48), with no significant differences among the subgroups. Participants with controlled glycaemia reported slightly higher scores than those with uncontrolled glycaemia (32 vs. 30, *p* = 0.367, Figure 2). Similarly, multivariable logistic regression did not identify a significant association between total MSPSS score and glycemic control (adjusted OR: 1.00, 95% CI: 0.98–1.04).

When analyzing different subscales of SS, family emerged as the most relevant subscale, with a median score of 12 out of 16 in the uncontrolled group and 13 in the controlled one. In contrast, support from friends was lower in the controlled group, with a median score of 6 vs. 7 out of 16, and exhibited greater variability. The importance of family support was reinforced in the multivariable regression, where it showed a positive association with GC (aOR: 1.07, 95% CI: 1.00–1.14), suggesting that for each unit increase in family support, the likelihood of achieving GC increased by 7%, after adjusting for sex, age, and diabetes duration (Table 2).

**Table 2:** Logistic regression analysis of well-controlled diabetes by source of social support.

Social support	OR crude	95% CI	OR adjusted*	95% CI
MSPSS total	1.01	(0.99, 1.04)	1.00	(0.98, 1.04)
MSPSS family	1.06	(0.99, 1.13)	**1.07**	**(1.00, 1.14)**
MSPSS friends	1.00	(0.95, 1.06)	1.00	(0.94, 1.06)
MSPSS other significant	1.01	(0.96, 1.06)	1.00	(0.95, 1.05)

*OR adjusted by sex, age and duration of diabetes. Bold values indicate statistically significant odds ratios (*p* < 0.05).

Overall, there was little variation in social support scores according to sociodemographic variables. Only friend support appeared lower among those without formal education (*p* = 0.047, Supplementary Table C).

However, sociodemographic differences were more visible in participants’ narratives, particularly regarding gender, where men and women described distinct patterns of support and coping. Quantitative findings also suggested a positive, though non-significant, association with “other significant” sources of support, which the qualitative interviews help to contextualize by illustrating contributions from neighbors, community groups, and patient clubs. Together, these qualitative insights enrich and deepen the understanding of the quantitative results, revealing how different sources of support are experienced and mobilized in everyday diabetes management.

### 3.2 Sources and type of social support perceived by participants

The qualitative sample of 16 interviewees was evenly split between men and women, aged 25–68, with most being older adults. There is ethnic, marital status, and employment diversity, alongside disparities in household income and family size (Table 3).

**Table 3:** Sociodemographic and clinical characteristics of participants with type 2 diabetes from the qualitative interviews (*N* = 16).

Participant ID	Sex	Age	Ethnicity	Marital status	Education level	Family members	Last year Occupation	Average monthly income ($)	T2DM duration (years)
P1*	Man	68	Mestizo	Married	Postgraduate	≥6	Unemployed	800	10–15
P2	Man	66	Black	Married	No formal schooling	2–3	Retired	466	10–15
P3	Woman	38	Mestizo	Single	Secondary	4–5	Self-employed	80–100	10–15
P4	Man	25	Mestizo	Single	University	≥6	Student	1,000	<5
P5	Woman	61	Mestizo	Married	Higher education	4–5	Community worker	1,500	>15
P6*	Woman	62	White	Single	Secondary	1	Homemaker	250	5–10
P7	Woman	47	Mulatto	Divorced	University	≥6	Public sector employee	900	<5
P8	Man	60	Mestizo	Married	Secondary	≥6	Unemployed	600	5–10
P9	Man	67	Montubio	Single	Primary	≥6	Unemployed	900	10–15
P10	Man	63	Indigenous	Married	Primary	2–3	Self-employed	300	10–15
P11	Woman	60	Mestizo	Married	University	4–5	Homemaker	1,000	>15
P12	Woman	65	Black	Married	Primary	2–3	Homemaker	400	10–15
P13	Man	42	Mestizo	Single	Secondary	2–3	Unemployed	600	10–15
P14	Woman	60	Mestizo	Married	University	2–3	Self-employed	4,000	>15
P15	Man	53	Mestizo	Married	Higher education	≥6	Self-employed	NA^a^ (high)	10–15
P16	Woman	63	Indigenous	Married	Primary	≥6	Self-employed	300	10–15

*Both migrants were from Venezuela, the rest Ecuadorians.

a NA, not answered with numeric details.

#### 3.2.1 Support from family

Emotional support from family emerged as a cornerstone of coping with diabetes, particularly during moments of emotional distress following diagnosis.

You know all the consequences that come with having this illness, right? And it's hard to accept… really hard to accept that you have it. I’m telling you, when my husband gave me the glucometer, it was like… like everything just came crashing down. Then, once it was clear I had the illness for good, they (my family) tried to lift my spirits. – P5 (Woman, 61)

Participants identified family support as a crucial motivator for treatment adherence and healthier behaviors, reinforcing self-discipline.

Sometimes when I’m about to eat something I shouldn’t, they make me realize and say, ‘You can’t eat that much, take care of yourself.’ Yeah, it’s important, because honestly, sometimes we really are tempted to eat things we shouldn’t. – P11 (Woman, 60)

Partners provided both emotional and instrumental support, this dynamic was particularly evident in couples where both partners had diabetes, as they supported each other and found it easier to adapt to behavioral changes together. However, economic constraints sometimes limited their ability to fully manage the condition.

We go for walks together, eat together, argue together… [Wife: I’ve got his blood pressure under control] … Everything. She checks my blood pressure every day — except for my sugar, because we haven’t been able to buy the device [the glucometer]. It’s pretty expensive. – P1 (Man, 68)

Two participants in a migration situation, both originally from Venezuela, highlighted the critical role of financial support from their families who had also migrated. One of them, living alone, expressed a desire to return to her family in Venezuela. However, this financial assistance was used as leverage to discourage her from going back to Venezuela, citing the lack of public healthcare resources necessary to manage her diabetes.

I have three children […] I came here because of them. One went to Mexico, another to the U.S., and the other one is in Venezuela. The one who supports me says, ‘Mom, you’re not going to Venezuela — the only thing you’d gain is being with family, but what about everything else? If you go to Venezuela, I won’t keep helping you.’ […] And my daughter says, ‘Mom, at least there in Ecuador you can see a doctor and get medicine… here, you can’t — not even if you have money to pay for it.’ – P6 (Woman, 62)

Many participants highlighted the adaptation of household dietary practices as crucial for diabetes management, with family acceptance of the changes providing practical and emotional support. This collective effort facilitated treatment adherence, improved household eating habits, and reinforced shared responsibility. Notably, these narratives were predominantly from women, reflecting their role as primary caregivers.

I cook for myself, and we all eat the same thing. So, there’s none of that ‘Oh, she’s on a diet, how annoying!’ No way! Everyone eats the same food and eats well. Everyone loses weight, we all keep ourselves in shape. Only my son and I have diabetes in our family of six. So, honestly, that’s a good thing, because I’ve seen friends whose families don’t support them. They make their own meals while the rest eat differently, and then they say, ‘Oh no.’ And they tell me, ‘I see them eating and it makes me want to eat too.’ But I don’t have that problem. – P7 (Woman, 47)

The lack of this understanding was a significant factor contributing to strained family relationships, as one woman explained:

He [her partner] knows I don’t cook for him like he eats at his mom’s. I’m not going to do that either because I have to be careful with my diabetes and my daughter’s health. Every Saturday, they have their barbecue — roasts, mayo, rotisserie chicken, pizzas… So honestly, it’s better if they don’t include me. I feel healthier and better that way. – P3 (Woman, 38)

Financial insecurity added to these challenges, often making it harder to follow the dietary recommendations needed for effective diabetes management.

My niece cooks, sometimes I do. We eat however we can, because we don’t have money, I don’t work, so we can’t really follow the diet properly. Of course, I know the diet needs to be stricter, but when there’s no money, there’s no way to do it. – P9 (Man, 67)

Beyond dietary support, participants frequently highlighted the practical support provided by family members. This included help with insulin injections, companionship during physical exercise such as walking, and financial support for basic needs, including food, living expenses, and purchasing medications during shortcuts in the public system.

My wife gives me my injections, or sometimes my daughter does when she’s around in the afternoon… as I have a visual impairment, they help me with it. – P8 (Man, 60)

Sometimes when the pills aren’t available here, my children help me. So, thank God my children help me. My children bring me little things, like ‘take, mum, have some fruit’, not sweet ones, but green pears. – P16 (Woman, 63)

However, a few participants expressed feelings of being a burden, reflecting the emotional strain of relying on others for financial or practical support in managing their diabetes. This perception often led to self-silencing and reluctance to seek help, further complicating the challenges of living with a chronic condition.

In this health centre, they help me a bit, but sometimes there aren’t any medicines. So, it’s really unfortunate. I mean, I can’t ask my wife for [money] either, right? because she’s the one who handles everything at home. So, things like food, basic bills, and all that are tough. That’s why, I tell you, asking my family for help is impossible. – P8 (Man, 60)

My cousin says ‘Eat what’s there darling, don’t say a word’, she says, ‘but don’t tell your mum, she has her own problems without taking on yours too’, it’s like adding another burden. – P3 (Woman, 38)

In contrast, others found strength and a sense of purpose in feeling useful within their support networks. For one woman, this sense of mutual care was particularly meaningful after her divorce:

As I said, I’m separated now. My husband is nowhere to be found. So, everyone stepped up to take care of me, and I repay them by looking after them, making meals and all that. […] So, I’m really happy. That’s why I don’t even feel the divorce, I don’t feel anything, because I’m surrounded by people who help me, who love me. And I think that’s the most important thing, having someone there to support you and not let you fall when you’re sick. – P7 (Woman, 47)

The role of family in informational support was less mentioned, but having a family member with diabetes provided valuable advice and also helped participants accept their diagnosis. Health professionals, however, remained the primary source of informational support.

M: For advice on managing diabetes, who do you usually turn to? I: Well, obviously the doctor first. Then my dad, since he’s been dealing with it [diabetes] for quite some time, he knows a lot about it. – P4 (Man, 25)

#### 3.2.2 Support from friends

Aligned with the findings from the MSPSS scores, interviewees generally mentioned weak friendship networks, particularly among men.

I have acquaintances, but no friends right now. – P13 (Man, 42)

Friends… No, there’s nothing. No support. No, I just live at home, I don’t go anywhere. – P9 (Man, 67)

Participants described the challenges of managing social pressure during social gatherings, where expectations often conflicted with their dietary restrictions. For example, difficulty of refusing certain foods or drinks without offending others, as well as frustration with friends who failed to respect their condition.

Here, when there are parties, they want to give me something sweet, and they immediately say, ‘no, no, no, don’t give C. any because she doesn’t eat it’. Sometimes I just take so they don’t feel bad. – P5 (Woman, 61)

When I was diagnosed, my friends acted like they just didn’t care… like an ‘I couldn’t care less’ attitude […] Some people even purposely say, ‘Have a glass of beer,’ but I can’t, beer is the worst for diabetes. So, what kind of friends are they, really? – P8 (Man, 60)

Male participants often associated their friendships with alcohol consumption, which became incompatible with diabetes-related restrictions. As a result, these friendships were lost, with men being ignored by their friends because they no longer drink, or the person with diabetes avoiding their friends because of social pressure to keep drinking.

The thing is, I don’t have many friends […] I used to be the loudest at parties, but since I’ve stopped drinking and smoking, it’s like the friendships have just faded away. I got to the point where I’d say ‘Hi, how are you?’ to my university mates and they’d just ignore me. That’s when I realized it was because I wasn’t drinking anymore. […] Sadly, I’ve been thinking about this a lot here in Ecuador and across Latin America, and there’s this silly idea that friendship only happens if there’s booze, partying, or a good time involved. – P15 (Man, 53)

The link between friendships and alcohol consumption was noted across all education levels, underscoring its cultural significance in the country. However, some participants described exceptions, highlighting friendships based on physical activities, such as sports or taking walks.

Ah, my football mates. They’re the ones I’m closest to, the ones I see the most each week, so to speak. We meet up for football, and sometimes we go out. – P4 (Man, 25)

A chilled game with colleagues. Like, I go out around 3 in the afternoon to play volleyball because the courts get really busy, loads of people there. So, me and my mates, friends, we all get together and have a laugh. It’s a good distraction for me… – P2 (Man, 66)

In a context of insecurity in streets and parks, particularly due to robbery, the lack of having someone to accompany them restricted their opportunities for outdoor physical activity.

I used to walk all the way to [a distant neighbourhood], but unfortunately, the lady who walked with me passed away, leaving me with no one to walk with, and I don’t risk going down there alone because it’s dangerous. – P5 (Woman, 61)

#### 3.2.3 Support from significant other/s

All participants taking part in a health-based support group emphasized its role in creating a sense of community (companionship) and offering practical tools for managing diabetes. These groups provide spaces for sharing experiences, having fun and relaxing, engaging in physical activities, and receiving informational support from health professionals and advice from their peers.

Being part of the diabetes group makes you feel less alone, knowing you’re not the only one dealing with this illness and that there are ways to manage it… That’s really important because not everyone has family support. – P14 (Woman, 60)

The group helps a lot because you actually have a bit of fun, and a good time at the club. Yes, they make you feel good there. We also go for capillary glucose tests […] And yes, we do a bit of exercise, play games, yeah, we do a lot of things. – P9 (Man, 67)

Time constraints, often driven by caregiving responsibilities for family members, particularly grandchildren, emerged as a significant barrier to participation in health-based support groups.

I no longer belong [to the club] because sometimes I need to look after my husband […] I go to drop my grandchildren off at school, I have a bit of a break there, and then I come back […] So, I don’t have enough time to be there. – P16 (Woman, 63)

Participants also identified community-based groups and social organisations, including sports clubs, urban gardening initiatives, traditional dance associations like ‘Los Negros de la Magdalena’, and religious groups such as ‘Cucuruchos.’^1^ These networks fostered a sense of belonging and facilitated close community ties, often serving as sources of social engagement, commitment, and recreation.

[The dance group] has helped me a lot [with diabetes] because it takes me away from all the stress completely, because you focus on it, and I try to give my best, you know? So, I pass that on to the kids and it helps me a lot. – P8 (Man, 60)

Work-related relationships, mentioned mostly by men, often dissolved after leaving or losing a job. However, one man explains how his friendships were sustained through labor unions, which provided a more lasting source of connection beyond the workplace.

M: And did you have a relationship with your work colleagues? I: Of course, we had been together for a few years, but now I haven’t seen them since I left, uh, I haven’t seen them anymore… – P9 (Man, 67)

With my former workmates, when we have the retirees’ union meeting, me and my old colleagues who worked together for more than 30 years, we get together every month, on the 15th. – P2 (Man, 66)

Less frequently, neighbors appeared as part of the extended network of support for some participants, providing companionship, sharing meals, and walking together. For example, one participant described her neighbor as a sister, highlighting the role of informal community ties in providing emotional and instrumental help.

I go for walks with my neighbor, [who I get on really well with] it’s great, wonderful. […] She’s attentive, when her daughter goes to work, she prepares her meals, like salad and grilled chicken, and she always saves some salad for me: ‘Here’s your salad, you do the rest.’ So, she shares with me because she knows I’ve got this diabetes problem. But it’s cool, she’s like a sister to me. – P6 (Woman, 62)

Interactions with healthcare professionals varied widely. Some participants reported positive experiences, showing the importance of informational support from doctors in improving their self-management. However, others highlighted challenges, including frequent changes in assigned doctors and perceived lack of interest from healthcare professionals.

They never examined me. As soon as I arrived, it was like, ‘Ah… you’re here for the medication, blah blah blah, here you go, off you go, buy the ice, pick it up at the pharmacy.’ – P15 (Man, 53)

#### 3.2.4 Conceptual framework

Overall, these narratives show the multifaceted nature of SS in diabetes management, showing how various formal and informal sources intersect to provide emotional, instrumental, and informational aid. Such support is vital for individuals managing diabetes and other chronic diseases, significantly influencing their ability to cope and maintain their wellbeing:

When you have support, you can get through it, above all, you can overcome it, and you can live, coexist with the illness. You learn to live with it. That’s the most important thing: having support. Whether it’s social, family, or medical, if you have that support, you can survive… – P7 (Woman, 47)

**Figure 3:**
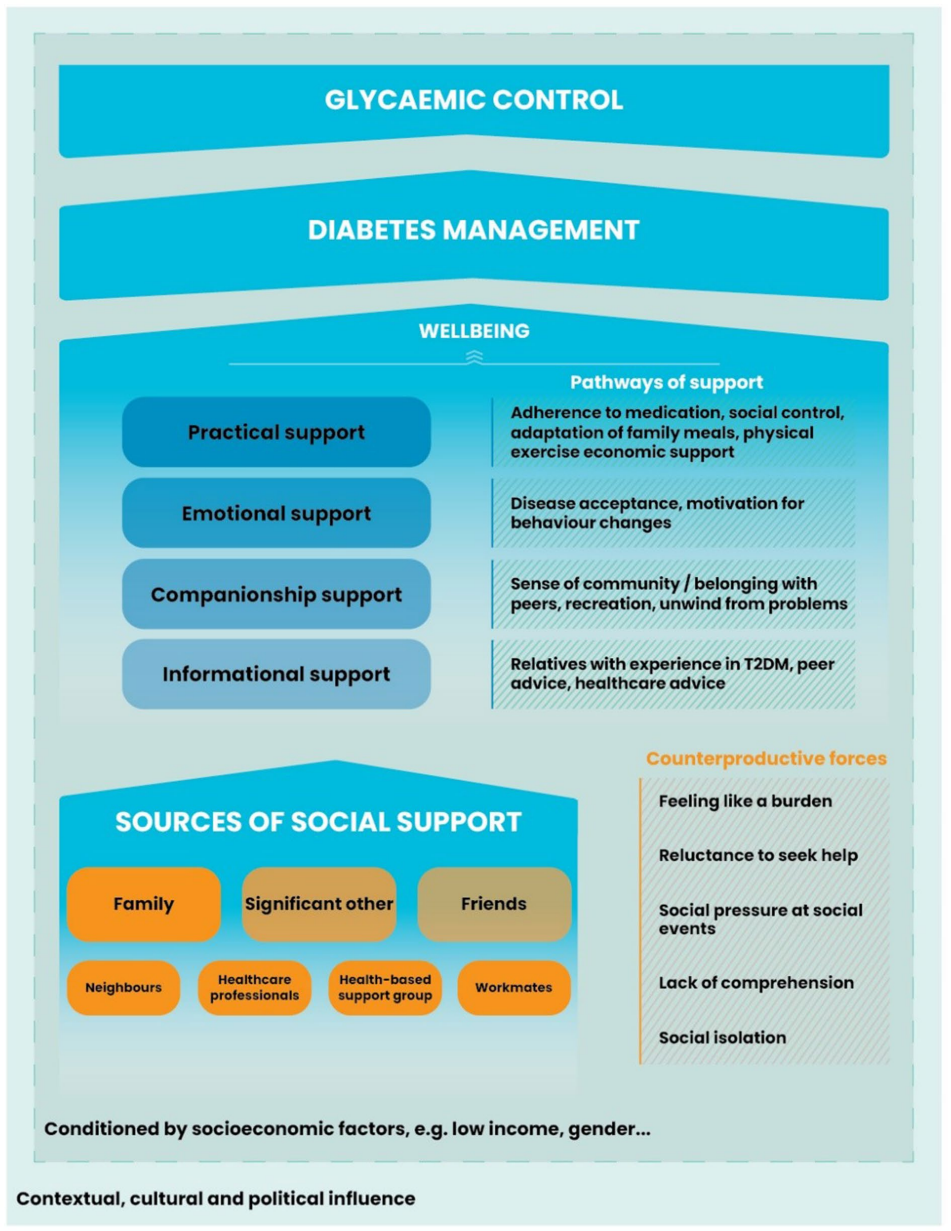
Conceptual framework of perceived social support influence on diabetes management and glycemic control.

Based on the participants’ accounts, we developed a conceptual framework to illustrate the main sources of social support in diabetes management and the multiple pathways through which this support may take form (Figure 3). While family remain a central pillar, other significant sources include neighbors, community groups, and patient clubs. In this low-income context, practical support emerged as particularly relevant: assisting with medication adherence, reinforcing health-promoting behaviors (social control), and facilitating their adoption. The figure also reflects counterproductive aspects of social support seen in the interviews, such as reluctance to seek help due to fear of being a burden, or social pressure during events. Overall, the figure summarizes how different types and sources of support interact along the pathway toward improved diabetes management and, ultimately, better GC.

## 4 Discussion

Our mixed-methods findings offer complementary insights into the role of social support in T2DM management within a low-income urban setting. While overall impact of perceived SS on GC was limited, family support consistently emerged as central factor, particularly highlighted by the qualitative findings. These narratives provide a deeper understanding of how emotional and instrumental support, alongside community networks, may enhance the everyday experiences of people living with T2DM.

Only 28.6% of participants achieved optimal glycemic control, a rate comparable to other low-income settings (1). Contrary to expectations, our study found no significant associations between GC and sociodemographic factors such as gender or age (33–35). This finding is consistent with other studies that have also failed to identify consistent sociodemographic predictors of GC (14), highlighting the complexity and context-dependence of these relationships. Among clinical variables, insulin users had poorer GC, likely reflecting indication bias (35, 36). Despite a high burden of comorbidities such as hypertension (67.5%), polymedication was not associated with worse GC, consistent with results from other local studies (36, 37).

Although total SS scores were not associated with GC, family support showed a modest yet positive association, a finding corroborated by the qualitative data. This aligns with literature emphasizing the vital role of family (38), in promoting self-care behaviors such as medication adherence, healthy eating, and regular monitoring (15, 39, 40). In contrast, friend support showed no association with GC; indeed, participants with optimal control reported lower scores in this domain, which alignes with previous studies in Latin America (41). This suggests that friendships may serve more as companionship rather than direct health-related assistance (42), and may be shaped by social norms, expectations and differences in how friendships are maintained in adulthood (43–45). The significant variation in friend support across education levels further supports this idea, as those with lower education reported less support from friends, reflecting broader social inequalities in the availability and quality of peer networks.

Gender dynamics provided further nuance to these findings. No gender differences emerged in SS scores, contrary to studies reporting a role in socialization and network dynamics (46–48). However, our qualitative data revealed that some men reported losing friendships after quitting alcohol due to diabetes care (49, 50), highlighting how male peer networks are often structured around alcohol-related practices (49, 50). In contrast, women emphasized family support and rarely mentioned friends, consistent with studies showing women’s greater reliance on household networks (51). These findings suggest that gender roles and social norms not only shape the type and quality of SS available but also influence its stability, with important implications for designing gender-sensitive interventions that strengthen alternative peer connections for men and leverage family-based support for women.

Participants also highlighted health-based support groups as beneficial for diabetes management (10, 52, 53), even though we observed no glycemic improvements among attendees, consistent with previous local research (54). Still, it is clear that belonging to a health-based support or community-based group can increase perception of SS and taking part in them may improve quality of life (55). Religion was also cited as a key source of motivation and purpose, with prayer and spiritual practices as effective coping mechanisms during periods of stress or health challenges, especially among people with less SS (56, 57).

While SS seems to play a crucial role, its benefits are contingent on broader socioeconomic conditions. In our study, financial constraints and healthcare inaccessibility potentially weakened support networks’ effectiveness, underscoring the need for structural interventions alongside social strategies (58). By recognizing that diabetes care extends beyond the individual and is influenced by broader social and economic factors, it becomes possible to develop more holistic strategies. Integrating SS into diabetes management efforts, such as community programs, patient support groups, or better-targeted services to individuals with low SS, could help reduce inequalities, improve wellbeing, and decrease the long-term burden of this increasingly common chronic disease. At the structural level, ministries of health could strengthen the recognition of social support and social determinants into chronic disease policies, while NGOs and local stakeholders could advocate for stronger social protection measures that sustain support networks. Embedding community and faith-based organizations within public health planning would further improve the integration of existing social resources, reframing diabetes management as a collective rather than individual responsibility.

This study has several limitations. The quantitative component relied on real-world data from clinical records in Quito’s public health system, which is affected by frequent administrative transitions, bureaucratic barriers, and fragmented service delivery. Despite approval from the Ministry of Health and an ethical board, incomplete or missing records hindered data extraction. Although we initially aimed to include a larger sample including individuals recruited at IESS facilities, inconsistencies and data loss that prevented confirmation of one-year follow-up in the IESS cohort.

The electronic registry system, while designed to standardize documentation, faces operational challenges. Health professionals described the system as slow and inefficient, contributing to incomplete or inconsistent records due to unstable internet connectivity, time constraints and administrative burden (25). These structural limitations contributed to incomplete data and reduced statistical power, particularly given the low prevalence of our primary outcome, optimal GC. Consequently, associations of modest effect size may have gone undetected.

In terms of data validity, several issues must be acknowledged: Absence of data in clinical records may not mean absence of medical attention, but limits our ability to assess the quality of care received. GC was defined using a combination of tests (HbA1c, fasting glucose, and capillary glucose), prioritizing HbA1c when available. This pragmatic approach may have led to classification bias.

The use of the MSPSS tool presents further limitations: it does not differentiate between types of support (practical, emotional, informational or companionship). While perceived and tangible support influence wellbeing, it remains unclear whether reported support translates into practical assistance, which could be particularly important in resource-constrained settings where access to diabetes care is challenging.

The “significant other” subscale scored high; however, a key limitation of the MSPSS is the lack of clarity regarding what this category represents. While it aims to capture the presence of a specific supportive person, its meaning can vary culturally. In Latin America, for example, it may be interpreted as a religious figure rather than a concrete social tie (28). As our study did not explore participants’ interpretations, this remains an open question.

Despite these limitations, the narratives of participants added contextual depth and helped contextualize the quantitative patterns, providing insight into the mechanisms through which social support shapes diabetes self-management in a fragmented health system. Although our study focused on a low-income urban community in Quito, the underlying dynamics, such as the centrality of family support, the context-specific constraints of peer networks, and the influence of socioeconomic conditions on the effectiveness of support, are likely relevant to other low- and middle-income settings, and can inform broader strategies across similar contexts.

## 5 Conclusion

Our findings highlight the pivotal role of family support in GC while emphasizing the broader potential of diverse social networks to enhance T2DM management. Interventions that integrate multiple sources of support, including peers, family, and health-based community groups, can provide emotional, practical, and informational aid, strengthening individuals’ coping capacities and overall wellbeing.

Future research should examine culturally adapted support interventions and validate social support measures in low-resource Latin American settings, while also exploring the qualitative aspects of social relationships to understand how different types of support influence self-care and how networks can be leveraged to improve diabetes outcomes across diverse sociodemographic contexts.

## Data Availability

The datasets presented in this study can be found in online repositories. The raw dataset from the retrospective cohort is available in Zenodo, indexed in OpenAIRE: https://doi.org/10.5281/zenodo.15005826.

## Footnotes

^1^Local religious organisations, often linked to Catholic communities, that coordinate social events, religious celebrations, and charitable activities to strengthen community bonds.

## References

[1] Sun H Saeedi P Karuranga S Pinkepank M Ogurtsova K Duncan BB . IDF diabetes Atlas: global, regional and country-level diabetes prevalence estimates for 2021 and projections for 2045. Diabetes Res Clin Pract. (2022) 183:109119. doi: 10.1016/j.diabres.2021.109119, PMID: 34879977 PMC11057359

[2] Altamirano Cordero L Vásquez M Cordero G Álvarez R Añez R Rojas J . Prevalence of type 2 diabetes mellitus and its risk factors in adult individuals from the city of Cuenca-Ecuador. Av Biomed. (2017) 6:10–21.

[3] Baldeón ME Felix C Fornasini M Zertuche F Largo C Paucar MJ . Prevalence of metabolic syndrome and diabetes mellitus type-2 and their association with intake of dairy and legume in Andean communities of Ecuador. PLoS One. (2021) 16:e0254812. doi: 10.1371/journal.pone.0254812 34297755 PMC8301611

[4] Orces CH Lorenzo C. Prevalence of prediabetes and diabetes among older adults in Ecuador: analysis of the SABE survey. Diabetes Metab Syndr Clin Res Rev. (2018) 12:147–53. doi: 10.1016/j.dsx.2017.12.002 29273428

[5] Puig-García M Caicedo-Montaño C Márquez-Figueroa M Chilet-Rosell E Montalvo-Villacis G Benazizi-Dahbi I . Prevalence and gender disparities of type 2 diabetes mellitus and obesity in Esmeraldas, Ecuador: a population-based survey in a hard-to-reach setting. Int J Equity Health. (2023) 22:1–12. doi: 10.1186/s12939-023-01939-x 37393298 PMC10314375

[6] Instituto Nacional de Estadística y Censos (INEC). Boletín Técnico Registro Estadístico de Defunciones Generales. Quito, Ecuador: Instituto Nacional de Estadística y Censos. (2022).

[7] Nicklett EJ Heisler MEM Spencer MS Rosland AM. Direct social support and long-term health among middle-aged and older adults with type 2 diabetes mellitus. J Gerontol B Psychol Sci Soc Sci. (2013) 68:933–43. doi: 10.1093/geronb/gbt100, PMID: 24150176 PMC3805290

[8] Strom JL Egede LE. The impact of social support on outcomes in adult patients with type 2 diabetes: a systematic review. Curr Diab Rep. (2012) 12:769–81. doi: 10.1007/s11892-012-0317-0, PMID: 22949135 PMC3490012

[9] Dale JR Williams SM Bowyer V. What is the effect of peer support on diabetes outcomes in adults? A systematic review. Diabet Med. (2012) 29:1361–77. doi: 10.1111/j.1464-5491.2012.03749.x 22804713

[10] Woodward A Walters K Davies N Nimmons D Protheroe J Chew-Graham CA . Barriers and facilitators of self-management of diabetes amongst people experiencing socioeconomic deprivation: a systematic review and qualitative synthesis. Health Expect. (2024) 27:e14070. doi: 10.1111/hex.14070 38751247 PMC11096776

[11] Keene DE Guo M Murillo S. “That wasn’t really a place to worry about diabetes”: housing access and diabetes self-management among low-income adults. Soc Sci Med. (2018) 197:71–7. doi: 10.1016/j.socscimed.2017.11.051, PMID: 29222997 PMC5771430

[12] Vanstone M Giacomini M Smith A Brundisini F DeJean D Winsor S. How diet modification challenges are magnified in vulnerable or marginalized people with diabetes and heart disease: a systematic review and qualitative meta-synthesis. Ont Health Technol Assess Ser. (2013) 13:1–40. PMC3817924 24228077

[13] Sallis J Owen N Fisher E. Ecological models of health behavior In: Glanz K Rimer B Viswanath K, editors. Health behavior: Theory, research, and practice. 4th ed. San Francisco, CA: Jossey-Bass (2008)

[14] Misra R Lager J. Ethnic and gender differences in psychosocial factors, glycemic control, and quality of life among adult type 2 diabetic patients. J Diabetes Complicat. (2009) 23:54–64. doi: 10.1016/j.jdiacomp.2007.11.003, PMID: 18413181

[15] Van Dam HA Van Der Horst FG Knoops L Ryckman RM Crebolder HFJM Van Den Borne BHW. Social support in diabetes: a systematic review of controlled intervention studies. Patient Educ Couns. (2005) 59:1–12. doi: 10.1016/j.pec.2004.11.001 16198213

[16] Baek RN Tanenbaum ML Gonzalez JS. Diabetes burden and diabetes distress: the buffering effect of social support. Ann Behav Med. (2014) 48:145–55. doi: 10.1007/s12160-013-9585-4, PMID: 24550072 PMC4249652

[17] Stopford R Winkley K Ismail K. Social support and glycemic control in type 2 diabetes: a systematic review of observational studies. Patient Educ Couns. (2013) 93:549–58. doi: 10.1016/j.pec.2013.08.016 24021417

[18] Kaplan RM Hartwell SL. Differential effects of social support and social network on physiological and social outcomes in men and women with type II diabetes mellitus. Health Psychol. (1987) 6:387–98. doi: 10.1037/0278-6133.6.5.387, PMID: 3678167

[19] Schram MT Assendelft WJJ van Tilburg TG Dukers-Muijrers NHTM. Social networks and type 2 diabetes: a narrative review. Diabetologia. (2021) 64:1905–16. doi: 10.1007/s00125-021-05496-2, PMID: 34189591 PMC8241411

[20] Blasco-Blasco M Puig-García M Piay N Lumbreras B Hernández-Aguado I Parker LA. Barriers and facilitators to successful management of type 2 diabetes mellitus in Latin America and the Caribbean: a systematic review. PLoS One. (2020) 15:e0237542. doi: 10.1371/journal.pone.0237542 32886663 PMC7473520

[21] Bernal-Soriano MC Barrera-Guarderas F Alonso-Jaquete A Chilet-Rosell E Benazizi I Caicedo-Montaño C . Contextualizing evidence for action on diabetes in low-resource settings-project CEAD part-II, strengthening the health system: a mixed-methods study protocol. Public Health. (2021) 18:18. doi: 10.3390/ijerph18073391, PMID: 33805911 PMC8037531

[22] O’cathain A Murphy E Nicholl J. The quality of mixed methods studies in health services research. J Health Serv Res Policy (2008) 1;13(2):92–98. doi: 10.1258/jhsrp.2007.007074 18416914

[23] Creswell JW Plano Clark VL. Designing and conducting mixed methods research. California: SAGE Publications (2007). 273 p.

[24] Lucio R Villacrés N Henríquez R. Sistema de salud de Ecuador. Salud Publica Mex. (2011) 53:177–87. Available online at: https://saludpublica.mx/index.php/spm/article/view/5039 21877083

[25] Bravo Díaz A Barrera Guarderas F Pinto Delgado J Torres AL Peralta A Blanes C . CEAD policy brief related to the implementation of comprehensive diabetes care in Quito. Ecuador: (2024).

[26] Zimet GD Dahlem NW Zimet SG Farley GK. The multidimensional scale of perceived social support. J Pers Assess. (1988) 52:30–41. doi: 10.1207/s15327752jpa5201_2 2280326

[27] Arechabala Mantuliz MC Miranda Castillo C. Validación de una escala de apoyo social percibido en un grupo de adultos mayores adscritos a un programa de hipertensión de la región metropolitana. Cienc Enferm. (2002) 8. doi: 10.4067/S0717-95532002000100007

[28] Ortiz Parada MS Baeza Rivera MJ. Propiedades psicométricas de una escala para medir apoyo social percibido en pacientes chilenos con diabetes tipo 2. Univ Psychol. (2010) 10:189–96. doi: 10.11144/Javeriana.upsy10-1.ppem

[29] Braun V Clarke V. To saturate or not to saturate? Questioning data saturation as a useful concept for thematic analysis and sample-size rationales. Qual Res Sport Exerc Health. (2021) 13:201–16. doi: 10.1080/2159676X.2019.1704846

[30] Lincoln YS Guba EG Pilotta JJ. Naturalistic inquiry. Int J Intercult Relat. (1985) 9:438–9.

[31] Shenton AK. Strategies for ensuring trustworthiness in qualitative research projects. Educ Inf. (2004) 22:63–75. doi: 10.3233/EFI-2004-22201

[32] Gale NK Heath G Cameron E Rashid S Redwood S. Using the framework method for the analysis of qualitative data in multi-disciplinary health research. BMC Med Res Methodol (2013);13:117. doi: 10.1186/1471-2288-13-117 24047204 PMC3848812

[33] Duarte G F, da Silva Moreira S da CC Almeida M de Souza Teles CA Andrade CS Reingold AL . Sex differences and correlates of poor glycaemic control in type 2 diabetes: a cross-sectional study in Brazil and Venezuela. BMJ Open (2019). doi: 10.1136/bmjopen-2018-023401, 9,:e023401 PMC6429715 30842107

[34] Eva IZ Ferdous C Qureshi NK Afrooz F. Gender-based disparities in glycemic control: insights from diabetes mellitus populations. Ann Int Med Dent Res. (2024) 10:67–73. doi: 10.53339/aimdr.2024.10.4.9

[35] Cheng LJ Wang W Lim ST Wu VX. Factors associated with glycaemic control in patients with diabetes mellitus: a systematic literature review. J Clin Nurs. (2019) 28:1433–50. doi: 10.1111/jocn.14795 30667583

[36] Urbina Carrera CA. Relación entre adherencia al tratamiento farmacológico y cifras de hb1ac en pacientes diabéticos pertenecientes al club de diabetes del Hospital General “Enrique Garcés” de quito en el año 2014. (2015)

[37] Rincón Alarcón A Gusñay Ramírez N Rodríguez VV. Adherencia Terapéutica En Pacientes Con Enfermedades Crónicas Del Club De Adultos Mayores De Un Centro De Salud, Ecuador. Anales de la Real Academia Nacional de Farmacia. (2020) 86:117–24. Avilable online at: https://efaidnbmnnnibpcajpcglclefindmkaj/https://analesranf.com/wp-content/uploads/2020/86_02/8602_04.pdf

[38] Camero Ortiz EA. A propósito del artículo “La atención en casa: El apoyo familiar en el control glicémico en pacientes con diabetes mellitus tipo 2.”. Hospital a Domicilio. (2024) 8:91–2. doi: 10.22585/hospdomic.v8i2.212

[39] Ortiz Claro YG Lindarte Clavijo AA Jiménez Sepúlveda MA Vega Angarita OM. Características sociodemográficas asociadas a la sobrecarga de los cuidadores de pacientes diabéticos en Cúcuta. Revista CUIDARTE. (2013) 4. doi: 10.15649/cuidarte.v4i1.5

[40] Martínez-Herrera E Moreno-Mattar O Dover RVH. El significado del capital social “individual” en diabéticos receptores de cuidado en un contexto urbano colombiano. Cad Saude Publica. (2015) 31:837–49. doi: 10.1590/0102-311X00167113 25945992

[41] Arteaga Noriega A Cogollo Jiménez R Muñoz Monterroza D. Apoyo social y control metabólico en la diabetes mellitus tipo 2. Revista CUIDARTE. (2017) 8:1668. doi: 10.15649/cuidarte.v8i2.405

[42] de la Rubia JM Alejandra Cerda MT. Predictores psicosociales de adherencia a la medicación en pacientes con diabetes tipo 2. Rev Iberoam Psicol Salud. (2015) 6:19–27. doi: 10.1016/S2171-2069(15)70003-7

[43] Penninx BWJH van Tilburg T Kriegsman DMW Boeke AJP Deeg DJH van Eijk JTM. Social network, social support, and loneliness in older persons with different chronic diseases. J Aging Health. (1999) 11:151–68. doi: 10.1177/089826439901100202, PMID: 10558434

[44] Fernández-Peña R Molina JL Valero O. Satisfaction with social support received from social relationships in cases of chronic pain: the influence of personal network characteristics in terms of structure, composition and functional content. Int J Environ Res Public Health. (2020) 17:2706. doi: 10.3390/ijerph17082706, PMID: 32326411 PMC7215382

[45] Shaw BA Krause N Liang J Bennett J. Tracking changes in social relations throughout late life. J Gerontol B Psychol Sci Soc Sci. (2007) 62:S90–9. doi: 10.1093/geronb/62.2.S90, PMID: 17379686

[46] Vaux A. Variations in social support associated with gender, ethnicity, and age. J Soc Issues (1985); 41(1):89–110. doi: 10.1111/j.1540-4560.1985.tb01118.x

[47] Matud MP Ibáñez I Bethencourt JM Marrero R Carballeira M. Structural gender differences in perceived social support. Pers Individ Dif. (2003) 35:1919–29. doi: 10.1016/S0191-8869(03)00041-2

[48] Rosenthal KR Gesten EL Shiffman S. Gender and sex role differences in the perception of social support. Sex Roles. (1986) 14:481–99. doi: 10.1007/BF00287449

[49] Emslie C Hunt K Lyons A. The role of alcohol in forging and maintaining friendships amongst Scottish men in midlife. Health Psychol. (2013) 32:33–41. doi: 10.1037/a0029874, PMID: 23316851

[50] Emslie C Hunt K Lyons A. Older and wiser? Men’s and women’s accounts of drinking in early mid-life. Sociol Health Illn. (2012) 34:481–96. doi: 10.1111/j.1467-9566.2011.01424.x, PMID: 22034902 PMC3491698

[51] Lynch SA. Who supports whom? How age and gender affect the perceived quality of support from family and friends. Gerontologist. (1998) 38:231–8. doi: 10.1093/geront/38.2.231, PMID: 9573668

[52] van Puffelen AL Rijken M Heijmans MJWM Nijpels G Schellevis FG. Effectiveness of a self-management support program for type 2 diabetes patients in the first years of illness: results from a randomized controlled trial. PLoS One. (2019) 14:e0218242. doi: 10.1371/journal.pone.0218242 31247039 PMC6597059

[53] Pienaar M Reid M. Self-management in face-to-face peer support for adults with type 2 diabetes living in low- or middle-income countries: a systematic review. BMC Public Health (2020);20:1834. doi: 10.1186/s12889-020-09954-1, PMID: 33256687 PMC7706053

[54] Aguinaga G Barrera F. Determinación de factores que afectan la adherencia al tratamiento en pacientes con Diabetes Mellitus 2, que acuden a un club de diabéticos. Rev Fac Cienc Médicas. (2014) 39:69–78. Available online at: https://revistadigital.uce.edu.ec/index.php/CIENCIAS_MEDICAS/article/view/1132/1132

[55] Heidi Lauckner Hutchinson S. Peer support for people with chronic conditions in rural areas: a scoping review. Rural Remote Health. (2016)16:3601 26943760

[56] Duke N. Type 2 diabetes self-management: spirituality, coping and responsibility. J Res Nurs. (2021) 26:743–60. doi: 10.1177/17449871211026958, PMID: 35251282 PMC8894753

[57] Ramírez Jiménez MG González-Arratia López-Fuentes NI Ruíz Martínez AO Van Barneveld HO Barcelata Eguiarte BE. Afrontamiento religioso y espiritualidad como mediadores entre estrés percibido y resiliencia en adultos con diabetes mellitus tipo 2. LIBERABIT Rev Peru Psicol (2022);28:e569. doi: 10.24265/liberabit.2022.v28n2.569

[58] Lewin AC Shamai M Novikov S. Surviving in crisis mode: the effect of material hardship and social support on emotional wellbeing among people in poverty during COVID-19. Soc Indic Res. (2023) 165:245–65. doi: 10.1007/s11205-022-03011-7, PMID: 36281265 PMC9581753

