# Supplementary material for: Untangling the role of social support in type 2 diabetes: insights from a mixed methods study in Quito, Ecuador
Source: Front Public Health. 2025 Nov 6;13:1668181. doi: 10.3389/fpubh.2025.1668181 (PMC12631610; doi:10.3389/fpubh.2025.1668181)
Supplement: Supplementary file 1 [file Supplementary_file_1.docx]

Supplementary Material

**Table A**: Sociodemographic and clinical characteristics of participants with type 2 diabetes from the cohort study considering glycaemic control in 3 categories (N=332).

| **Variable** | **Controlled**  **n (%)** | **Poorly Controlled**  **n (%)** | **Not Controlled**  **n (%)** | **Total**  **N (%)** | **p-value** |
| --- | --- | --- | --- | --- | --- |
| **Sex** |  |  |  |  | **0.503** |
| Men | 15 (24.6%) | 35 (57.4%) | 11 (18.0%) | 61 (100%) |  |
| Women | 80 (29.5%) | 133 (49.1%) | 58 (21.4%) | 271 (100%) |  |
| **Age**  median (IQR) years | 63 (55–72) | 63.5 (58–70.5) | 67 (59–74) | 64 (58–71.5) | **0.408** |
| **Ethnicity** |  |  |  |  | **0.086** |
| Mestizo | 85 (29.3%) | 150 (51.7%) | 55 (19.0%) | 290 (100%) |  |
| White | 8 (34.8%) | 6 (26.1%) | 9 (39.1%) | 23 (100%) |  |
| Afro | 0 (0.0%) | 3 (60.0%) | 2 (40.0%) | 5 (100%) |  |
| Indigenous | 2 (16.7%) | 8 (66.7%) | 2 (16.7%) | 12 (100%) |  |
| **Education Level** |  |  |  |  | **0.438** |
| No formal schooling | 8 (25.0%) | 17 (53.1%) | 7 (21.9%) | 32 (100%) |  |
| Primary school | 53 (27.5%) | 104 (53.9%) | 36 (18.6%) | 193 (100%) |  |
| Secondary school | 23 (29.5%) | 33 (42.3%) | 22 (28.2%) | 78 (100%) |  |
| Higher education | 11 (37.9%) | 14 (48.3%) | 4 (13.8%) | 29 (100%) |  |
| **Civil Status** |  |  |  |  | **0.560** |
| Married | 42 (26.4%) | 84 (52.8%) | 33 (20.8%) | 159 (100%) |  |
| Separated/divorced | 20 (33.9%) | 31 (52.5%) | 8 (13.6%) | 59 (100%) |  |
| Single | 14 (33.3%) | 16 (38.1%) | 12 (28.6%) | 42 (100%) |  |
| Widowed | 12 (23.5%) | 26 (51.0%) | 13 (25.5%) | 51 (100%) |  |
| Living with partner | 6 (30.0%) | 11 (55.0%) | 3 (15.0%) | 20 (100%) |  |
| **Employment Status** |  |  |  |  | **0.167** |
| Unpaid/Other | 43 (26.5%) | 78 (48.1%) | 41 (25.3%) | 162 (100%) |  |
| Self-employed | 38 (29.0%) | 68 (51.9%) | 25 (19.1%) | 131 (100%) |  |
| Employed | 14 (35.9%) | 22 (56.4%) | 3 (7.7%) | 39 (100%) |  |
| **Household Earnings** |  |  |  |  | **0.578** |
| <$375 | 45 (29.6%) | 78 (51.3%) | 29 (19.1%) | 152 (100%) |  |
| ≥$375 | 34 (30.4%) | 51 (45.5%) | 27 (24.1%) | 112 (100%) |  |
| NS/NC | 16 (23.5%) | 39 (57.4%) | 13 (19.1%) | 68 (100%) |  |
| **Duration of T2DM** median (IQR) years | 10 (6–17) | 12 (6–19) | 11.5 (5–16) | 11 (6–18) | **0.152** |
| **Type of Treatment** |  |  |  |  | **<0.001** |
| No treatment | 4 (30.8%) | 1 (7.7%) | 8 (61.5%) | 13 (100%) |  |
| Only oral meds | 59 (36.4%) | 67 (41.4%) | 36 (22.2%) | 162 (100%) |  |
| Only insulin | 4 (18.2%) | 11 (50.0%) | 7 (31.8%) | 22 (100%) |  |
| Oral + insulin | 28 (20.7%) | 89 (65.9%) | 18 (13.3%) | 135 (100%) |  |
| **Polipharmacy** |  |  |  |  | **<0.001** |
| Less than 3 meds | 40 (29.0%) | 55 (39.9%) | 43 (31.2%) | 138 (100%) |  |
| ≥3 meds | 55 (28.4%) | 113 (58.2%) | 26 (13.4%) | 194 (100%) |  |
| **Belongs to Health-Based Support Group** |  |  |  |  | **0.435** |
| No | 67 (29.7%) | 109 (48.2%) | 50 (22.1%) | 226 (100%) |  |
| Yes | 28 (26.4%) | 59 (55.7%) | 19 (17.9%) | 106 (100%) |  |
| **Total** | **95 (28.6%)** | **168 (50.6%)** | **69 (20.8%)** | **332 (100%)** |  |

**Table B**: Clinical picture of complications/comorbidities (N=332).

| Complications / Comorbidities | Total  n (%) |
| --- | --- |
| Hypertension | 224 (67.5%) |
| Dyslipidaemia | 143 (43.1%) |
| Retinopathy | 43 (13.0%) |
| Dental Disease | 38 (11.5%) |
| Kidney Disease | 20 (6.0%) |
| Diabetic Foot | 14 (4.2%) |
| Erectile Dysfunction* | 1 (1.6%) |
| Amputation | 1 (0.3%) |
| Total | **332 (100%)** |

*n total = 61 men. No differences by sex.

**Table C:** Sociodemographic characteristics stratified by social support subscales (MSPSS scores), (N=332).

| MSPSS score | **Total (12-48)**  **Median (IQR)** | **p-value†** | **Family (4-16)**  **Median (IQR)** | **p-value†** | **Friends (4-16)**  **Median (IQR)** | **p-value†** | **Other Significant**  **Median (IQR)** | **p-value†** |
| --- | --- | --- | --- | --- | --- | --- | --- | --- |
| **Sex** |  | 0.211 |  | 0.230 |  | 0.488 |  | 0.728 |
| Men | 28 (20–36) |  | 11 (7–14) |  | 6 (4–11) |  | 11 (5–16) |  |
| Women | 31 (24–36) |  | 13 (9–15) |  | 7 (4–12) |  | 12 (6–16) |  |
| **Age** |  | 0.868 |  | 0.439 |  | 0.506 |  | 0.204 |
| < 65 years | 31 (23–36) |  | 12 (8–15) |  | 6 (4–12) |  | 12 (7–16) |  |
| ≥ 65 years | 31 (23–37) |  | 13 (9–15) |  | 7 (4–12) |  | 11 (6–16) |  |
| **Ethnicity‡** |  | 0.773 |  | 0.569 |  | 0.713 |  | 0.535 |
| Mestizo | 31 (23–36) |  | 12 (8–15) |  | 7 (4–12) |  | 12 (6–16) |  |
| White | 25 (19–37) |  | 11 (8–14) |  | 8 (4–12) |  | 8 (4–16) |  |
| Afro | 28 (22–39) |  | 13 (7–14) |  | 8 (4–16) |  | 4 (4–16) |  |
| Indigenous | 31 (22–34) |  | 11 (7–14) |  | 5 (4–10) |  | 12 (7–15) |  |
| **Education** |  | 0.183 |  | 0.423 |  | **0.047** |  | 0.829 |
| No formal schooling | 29 (22–35) |  | 11 (8–14) |  | 5 (4–8) |  | 10 (7–15) |  |
| Primary school | 31 (23–36) |  | 13 (9–15) |  | 6 (4–11) |  | 12 (6–16) |  |
| Secondary school | 32 (24–38) |  | 13 (8–14) |  | 8 (4–12) |  | 12 (6–16) |  |
| Higher education | 33 (24–37) |  | 11 (8–14) |  | 7 (4–15) |  | 12 (6–16) |  |
| **Civil Status‡** |  | 0.720 |  | 0.520 |  | 0.414 |  | 0.276 |
| Married | 32 (23–36) |  | 12 (9–15) |  | 6 (4–11) |  | 12 (7–16) |  |
| Separated or divorced | 32 (21–41) |  | 12 (7–14) |  | 7 (4–14) |  | 12 (6–16) |  |
| Single | 29 (24–35) |  | 13 (7–15) |  | 7 (4–10) |  | 11 (5–14) |  |
| Widowed | 31 (24–36) |  | 12 (9–14) |  | 8 (4–13) |  | 12 (4–15) |  |
| Living with partner | 28 (22–34) |  | 11 (7–14) |  | 7 (4–11) |  | 11 (4–15) |  |
| **Employment** |  | 0.752 |  | 0.756 |  | 0.902 |  | 0.607 |
| Unpaid | 31 (24–36) |  | 13 (9–15) |  | 7 (4–11) |  | 12 (6–16) |  |
| Self-employed | 31 (22–36) |  | 12 (8–15) |  | 6 (4–12) |  | 12 (6–16) |  |
| Employed | 31 (24–38) |  | 12 (8–14) |  | 6 (4–12) |  | 12 (8–16) |  |
| **Income** |  | 0.411 |  | 0.398 |  | 0.573 |  | 0.244 |
| <$375 | 31 (22–36) |  | 12 (8–14) |  | 8 (4–12) |  | 12 (6–16) |  |
| ≥$375 | 31 (25–38) |  | 12 (9–15) |  | 7 (4–12) |  | 12 (7–16) |  |
| NS/NC | 30 (22–36) |  | 13 (9–15) |  | 6 (4–10) |  | 11 (4–16) |  |
| **Duration of DM‡** |  | 0.591 |  | 0.457 |  | 0.596 |  | 0.368 |
| < 10 years | 32 (23–37) |  | 12 (8–15) |  | 7 (4–12) |  | 12 (7–16) |  |
| ≥ 10 years | 31 (22–36) |  | 13 (8–15) |  | 7 (4–11) |  | 11 (6–16) |  |
| **Belongs to support Group** |  | <0.001 |  | <0.001 |  | <0.001 |  | 0.133 |
| No | 29 (22–35) |  | 12 (7–14) |  | 5 (4–10) |  | 12 (6–16) |  |
| Yes | 34 (26–41) |  | 13 (10–15) |  | 9 (6–12) |  | 12 (7–16) |  |

**†**p value <0.05 (Kwallis test); **‡**2 missing ethnicity; 1 missing civil status, 5 missing T2DM duration.
