# Supplementary material for: Untangling the role of social support in type 2 diabetes: insights from a mixed methods study in Quito, Ecuador
Source: Front Public Health. 2025 Nov 6;13:1668181. doi: 10.3389/fpubh.2025.1668181 (PMC12631610; doi:10.3389/fpubh.2025.1668181)
Supplement: Supplementary file 2 [file Supplementary_file_2.docx]

Supplementary Material 2

Supplementary table: Translation of the transcript’s quotes.

| ***Original [Spanish]*** | ***Translated [English]*** |
| --- | --- |
| *Uno sabe todas las consecuencias que conlleva el tener esa enfermedad ¿no?, y es difícil aceptar… es muy difícil aceptar que usted tiene la enfermedad. Ya le digo, cuando mi esposo me vino a dar el glucómetro fue como que…. como que se me derrumbó todo. Ya pues ya cuando estuve con la enfermedad para siempre como que [mis familiares] trataron de alzarme mi autoestima. – P5 (Mujer, 61)* | You know all the consequences that come with having this illness, right? And it's hard to accept… really hard to accept that you have it. I’m telling you, when my husband gave me the glucometer, it was like… like everything just came crashing down. Then, once it was clear I had the illness for good, they [my family] tried to lift my spirits. – P5 (Woman, 61) |
| *A veces cuando estoy por comer algo que no me corresponde, me hacen caer en cuenta y me dicen, “no puedes comer tanto, cuídate”. Sí, sí, es importante, porque en realidad a veces, si estamos tentados a comer lo que no deberíamos. – P11 (Mujer, 60)* | Sometimes when I’m about to eat something I shouldn’t, they make me realize and say, ‘You can’t eat that much, take care of yourself.’ Yeah, it’s important, because honestly, sometimes we really are tempted to eat things we shouldn’t. – P11 (Woman, 60) |
| *Salimos a caminar juntos, comemos juntos, peleamos juntos... [Esposa: Ya le tengo controladita la presión.] …Todo, todos los días me toma la tensión, menos la de la azúcar, porque no hemos podido comprarla por el aparatico [glucómetro] que sí es caro. – P1 (Hombre, 68)* | We go for walks together, eat together, argue together… [Wife: I’ve got his blood pressure under control]… Everything. She checks my blood pressure every day — except for my sugar, because we haven’t been able to buy the device [the glucometer]. It’s pretty expensive. – P1 (Man, 68) |
| *Yo tengo tres hijos […] yo me vine para acá por ellos. Uno se me fue para México, el otro se me fue para los Estados Unidos y la otra está en Venezuela. Entonces, el que me mantiene me dice “mamá, tú no te vas ir para Venezuela, porque la única ventaja que tú vas a tener es que vas a estar con tu familia, pero ¿y lo demás? Si tú estás para Venezuela, yo no te sigo ayudando,” dice él... […] Mi hija me dice, “mamá, allá [Ecuador] por lo menos tú percibes médico y percibes medicina... aquí... no la consigues, así tengas [dinero] para comprarla.” – P6 (Mujer, 62)* | I have three children […] I came here because of them. One went to Mexico, another to the U.S., and the other one is in Venezuela. The one who supports me says, ‘Mom, you’re not going to Venezuela — the only thing you’d gain is being with family, but what about everything else? If you go to Venezuela, I won’t keep helping you.’ […] And my daughter says, ‘Mom, at least there in Ecuador you can see a doctor and get medicine… here, you can’t — not even if you have money to pay for it.’ – P6 (Woman, 62) |
| *Yo cocino para mí y todos comemos igual. Entonces, no hay eso de que: “ay, no ella está haciendo dieta, que feo” ¡No! Todos comen lo mismo y comen bien, baja de peso todo el mundo, todos nos mantenemos. Solo mi hijo y yo somos los diabéticos en la casa de seis. Entonces ya le digo, es lo bueno, porque yo he visto a amigas que a ellas la familia no les apoya. Ellas hacen su comida y dicen que el resto comen y le dicen: “ay, no” Y ellas dicen: “yo les veo comer a ellos y me da ganas de comer.” Entonces, yo no tengo eso. – P7 (Mujer, 47)* | I cook for myself, and we all eat the same thing. So there’s none of that ‘Oh, she’s on a diet, how annoying!’ No way! Everyone eats the same food and eats well. Everyone loses weight, we all keep ourselves in shape. Only my son and I have diabetes in our family of six. So, honestly, that’s a good thing, because I’ve seen friends whose families don’t support them. They make their own meals while the rest eat differently, and then they say, ‘Oh no.’ And they tell me, ‘I see them eating and it makes me want to eat too.’ But I don’t have that problem. – P7 (Woman, 47) |
| *Él [su pareja] sabe que no le doy de comer, así como él come donde su mamá. Yo no voy a hacer eso tampoco, porque tengo que tener cuidado por mi diabetes y la salud de mi hija. Ellos cada sábado es ya su parrillada, sus asados, sus mayonesas, su pollo roster, sus pizzas… Entonces a mí no mejor, para mí, que no me tomen en cuenta, yo me siento mejor saludable y bien. – P3 (Mujer, 38)* | He [her partner] knows I don’t cook for him like he eats at his mom’s. I’m not going to do that either because I have to be careful with my diabetes and my daughter’s health. Every Saturday, they have their barbecue — roasts, mayo, rotisserie chicken, pizzas… So honestly, it’s better if they don’t include me. I feel healthier and better that way. – P3 (Woman, 38) |
| *Cocina mi sobrina, a veces yo. La alimentación se hace cómo se puede, porque como hace falta el dinero, por lo que no trabajo, no se puede seguir la dieta como debe de ser pues. Claro, lo sé que la dieta es más estricta, pero cuando no hay, no hay cómo. – P9 (Hombre, 67)* | My niece cooks, sometimes I do. We eat however we can, because we don’t have money, I don’t work, so we can’t really follow the diet properly. Of course, I know the diet needs to be stricter, but when there’s no money, there’s no way to do it. – P9 (Man, 67) |
| *Mi esposa me inyecta, o a veces mi hija cuando está en la tarde… como yo tengo la discapacidad visual entonces ellas me inyectan, es la ayuda de ellas. – P8 (Hombre, 60)* | My wife gives me my injections, or sometimes my daughter does when she’s around in the afternoon… as I have a visual impairment, they help me with it. – P8 (Man, 60) |
| *A veces cuando aquí no hay las pastillas, mis hijos me ayudan. Entonces por ahí gracias a Dios que me ayudan mis hijos. Mis hijos así… cualquier cosita, me traen ‘tome mamá, coma una frutita’, así que no es con dulce, sino esas peras verdes. – P16 (Mujer, 63)* | Sometimes when the pills aren’t available here, my children help me. So, thank God my children help me. My children bring me little things, like ‘take, mum, have some fruit’, not sweet ones, but green pears. – P16 (Woman, 63) |
| *En este centro de salud me ayudan algo, pero hay veces que no hay medicinas. Entonces es muy lamentable, le digo, o sea, yo tampoco puedo decirle a mi mujer que me dé [dinero] ¿no?, porque ella es la que afronta toda la casa. Entonces si es duro la alimentación, pagos básicos y todo eso. Tonces’ eso le digo, de ahí [pedir] ayuda a la familia imposible. – P8 (Hombre, 60)* | In this health centre, they help me a bit, but sometimes there aren’t any medicines. So, it’s really unfortunate. I mean, I can’t ask my wife for [money] either, right? because she’s the one who handles everything at home. So, things like food, basic bills, and all that are tough. That’s why, I tell you, asking my family for help is impossible. – P8 (Man, 60) |
| *Mi prima dice ‘come lo que hay mija, no hables ni digas nada,’ dice, ‘pero ahora [no le digas] tu mamá ni nada,’ dice ‘ella también tiene sus propios problemas como para estar con los tuyos más’, o sea es una carga más. – P3 (Mujer, 38]* | My cousin says ‘Eat what’s there *darling*, don’t say a word’, she says, ‘but don’t tell your mum, she has her own problems without taking on yours too’, it’s like adding another burden. – P3 (Woman, 38] |
| *Como le digo, estoy separada ahorita. Mi marido brilla por la ausencia. Entonces, todo el mundo se hizo a cargo de mí y yo retribuyo cuidándoles así, así, [preparando] la comida y todo. [...] Entonces yo también qué felicidad. Por eso, no siento ni el divorcio, no siento nada, porque estoy ahí acompañada, estoy con la gente que me ayuda, que me quiere, que yo creo que eso es lo más importante. Que haya quien a uno le ayude y no le deje el rato de una enfermedad caer. – P7 (Mujer, 47)* | As I said, I’m separated now. My husband is nowhere to be found. So, everyone stepped up to take care of me, and I repay them by looking after them, making meals and all that. […] So, I’m really happy. That’s why I don’t even feel the divorce, I don’t feel anything, because I’m surrounded by people who help me, who love me. And I think that’s the most important thing, having someone there to support you and not let you fall when you’re sick.– P7 (Woman, 47) |
| *M: Para consejo sobre el manejo de la diabetes, ¿a quién sueles acudir?*  *E: Bueno, en la parte específica al doctor, obviamente. De ahí a mi papá, que él como ha estado bastante tiempo con esto [la diabetes], ya sabe. – P4 (Hombre, 25)* | M: For advice on managing diabetes, who do you usually turn to?  I: Well, obviously the doctor first. Then my dad, since he’s been dealing with it [diabetes] for quite some time, he knows a lot about it. – P4 (Man, 25) |
| *Tengo conocidos, amistades en este tiempo no hay. – P13 (Hombre, 42)* | I have acquaintances, but no friends right now. – P13 (Man, 42) |
| *Amigos... No, no hay nada. No hay apoyo. No, solo vivo en la casa, no salgo para ningún lado. – P9 (Hombre, 67)* | Friends… No, there’s nothing. No support. No, I just live at home, I don’t go anywhere. – P9 (Man, 67) |
| *Aquí cuando hay las fiestas, me quieren dar algo dulce, y ya me vienen a dar: ‘no, no, no, a C. no le den porque ella no come’. A veces si les cojo es por no hacerles sentir mal. – P5 (Mujer, 61)* | Here, when there are parties, they want to give me something sweet, and they immediately say, ‘no, no, no, don’t give C. any because she doesn’t eat it’. Sometimes I just take so they don’t feel bad.– P5 (Woman, 61) |
| *El diagnóstico de la enfermedad para mis amistades fue, como decir…, un ‘quemeimportismo’ […] Hay gente que a veces parece de adrede te dice ‘sírvete un vaso de cerveza’, no puedo, si la cerveza es la más mortal para la diabetes, tons’ qué clase de amigos son ¿no? – P8 (Hombre, 60)* | When I was diagnosed, my friends acted like they just didn’t care… like an ‘I couldn’t care less’ attitude […] Some people even purposely say, ‘Have a glass of beer,’ but I can’t, beer is the worst for diabetes. So, what kind of friends are they, really?– P8 (Man, 60) |
| *Lo que pasa es que soy poco de amigos […] Antes, si era él que más gritaba, porque me iba a las fiestas, pero a raíz de que ya no tomo, no fumo, entonces es como que los amigos ya no, o sea, ya la amistad, o sea, llegó a un punto de que saludaba a mis colegas, compañeros de universidad… de ‘Hola, ¿cómo estás?’ y me dejaban en visto, entonces me di cuenta de que era por lo que no tomaba. [...] Lamentablemente yo me he puesto a analizar a nivel de Ecuador y a nivel de Latinoamérica tenemos ese concepto tan... tan torpe de la... de que si... para ser amistad, para hacer todo si no hay licor, no hay fiesta o gozo... – P15 (Hombre, 53)* | The thing is, I don’t have many friends […] I used to be the loudest at parties, but since I’ve stopped drinking and smoking, it’s like the friendships have just faded away. I got to the point where I’d say ‘Hi, how are you?’ to my university mates and they’d just ignore me. That’s when I realized it was because I wasn’t drinking anymore. […] Sadly, I’ve been thinking about this a lot here in Ecuador and across Latin America, and there’s this silly idea that friendship only happens if there’s booze, partying, or a good time involved. – P15 (Man, 53) |
| *Ah, mis amigos del fútbol. Ellos son los que más con los que más llevo relación, con los que más veces me junto a la semana, por decirlo de una forma. Quedamos para el fútbol, para salir algunas veces. – P4 (Hombre, 25)* | Ah, my football mates. They’re the ones I’m closest to, the ones I see the most each week, so to speak. We meet up for football, and sometimes we go out. – P4 (Man, 25) |
| *Un juego así tranquilo con los compañeros. Así sea, por ejemplo, yo salgo, por ejemplo, las 3 de la tarde y aquí, al voley, porque ahí se llenan las canchas ahí atrás, entonces hay bastante gente, entonces ahí entre compañeros, amigos, y todo eso ahí nos agrupamos, estamos así. Me distraigo todo eso... – P2 (Hombre, 66)* | A chilled game with colleagues. Like, I go out around 3 in the afternoon to play volleyball because the courts get really busy, loads of people there. So, me and my mates, friends, we all get together and have a laugh. It’s a good distraction for me... – P2 (Man, 66) |
| *Yo me iba hasta [un barrio lejos] caminando, pero para mala suerte la señora que caminaba conmigo se murió, ya me quedé sin nadie para caminar, y solita no me arriesgó a irme para abajo, porque sí es peligroso. – P5 (Mujer, 61)* | I used to walk all the way to [a distant neighborhood], but unfortunately, the lady who walked with me passed away, leaving me with no one to walk with, and I don’t risk going down there alone because it’s dangerous. – P5 (Woman, 61) |
| *E: Participar en el grupo de diabetes hace sentirse acompañado, el saber que no es el único con esta enfermedad y que hay posibilidades de sobrellevar este problema... entonces eso es muy importante porque no todo el mundo tiene el apoyo de su familia. – P14 (Mujer, 60)* | Being part of the diabetes group makes you feel less alone, knowing you’re not the only one dealing with this illness and that there are ways to manage it... That’s really important because not everyone has family support. – P14 (Woman, 60) |
| *El grupo ayuda bastante pues, porque uno sí se divierte un poquito, pasarla bien también ahí en el club. Sí le hacen sentir bien ahí. También acudimos a tomar la glucosa capilar […] Sí, hacemos un poco de ejercicio, por ahí juegos, sí, se hace bastante cosas. – P9 (Hombre, 67)* | The group helps a lot because you actually have a bit of fun, and a good time at the club. Yes, they make you feel good there. We also go for capillary glucose tests […] And yes, we do a bit of exercise, play games, yeah, we do a lot of things. – P9 (Man, 67) |
| *Yo ya no pertenezco [al club] porque es que tengo que a veces asearle a mi marido. [...] Me voy a dejar a mis nietos en la escuela, me distraigo ahí, y regreso […] Entonces, no tengo el tiempo suficiente para estar. – P16 (Mujer, 63)* | I no longer belong [to the club] because sometimes I need to look after my husband [...] I go to drop my grandchildren off at school, I have a bit of a break there, and then I come back […] So, I don’t have enough time to be there. – P16 (Woman, 63) |
| *E: [El grupo de danza]…me ha ayudado bastante [con la diabetes], porque me salva de todo el estrés totalmente, porque usted se enfoca en eso, y entonces trato de dar lo mejor de todo ¿no?. Entonces eso les inculcó a los chicos y me ayuda bastante. – P8 (Hombre, 60)* | I: [The dance group] has helped me a lot [with diabetes] because it takes me away from all the stress completely, because you focus on it, and I try to give my best, you know? So, I pass that on to the kids and it helps me a lot. – P8 (Man, 60) |
| *M: ¿Y tenía relación con sus compañeros de trabajo?*  *E: Claro, que ya tenía unos años juntos, ahorita si ya no los he visto desde que salí, uh ya no les vi más... – P9 (Hombre, 67)* | M: And did you have a relationship with your work colleagues?  I: Of course, we had been together for a few years, but now I haven’t seen them since I left, uh, I haven’t seen them anymore… – P9 (Man, 67) |
| *Con los compañeros de trabajo, que cuando tenemos la reunión del sindicato de jubilados que tenemos con mis compañeros antiguos que fuimos trabajadores por más de 30 años que, de ahí, entonces con ellos nos topamos cada mes, cada 15 de cada mes– P2 (Hombre, 66)* | With my former workmates, when we have the retirees’ union meeting, me and my old colleagues who worked together for more than 30 years, we get together every month, on the 15th. – P2 (Man, 66) |
| *Salgo a caminar con la vecina [con quien me llevo] chévere, de maravilla. […] Ella está pendiente, cuando la hija se va por el trabajo, ella le prepara su comida, su ensalada, su pollo a la plancha, y ella me guarda siempre su ensalada: ‘Aquí tiene ensalada, usted hace lo demás’. O sea, ella comparte conmigo porque sabe que yo tengo ese problema de diabetes. Pero es chévere, es como si fuera una hermana para mí. – P6 (Mujer, 62)* | I go for walks with my neighbor, [who I get on really well with] it’s great, wonderful. […] She’s attentive, when her daughter goes to work, she prepares her meals, like salad and grilled chicken, and she always saves some salad for me: ‘Here’s your salad, you do the rest.’ So, she shares with me because she knows I’ve got this diabetes problem. But it’s cool, she’s like a sister to me. – P6 (Woman, 62) |
| *Nunca me revisaban, sino que, a lo que llegaban, era ‘ah… usted viene por la medicación, ta-ta, ta-ta, tenga, vaya, retire rápido, compre el hielo, retire en la farmacia’. – P15 (Hombre, 53)* | They never examined me. As soon as I arrived, it was like, ‘Ah... you’re here for the medication, *blah blah* *blah,* here you go, off you go, buy the ice, pick it up at the pharmacy.’ – P15 (Man, 53) |
| *Cuando uno tiene apoyo, se puede salir, más que todo, se puede superar, y se puede vivir, convivir, con la enfermedad. Se aprende a vivir con la enfermedad. Es lo más importante, de tener el apoyo. Ya sea social, ya sea familiar, o como digo, médicos, si uno tiene ese apoyo, se puede sobrevivir... – P7 (Mujer, 47)* | When you have support, you can get through it, above all, you can overcome it, and you can live, coexist with the illness. You learn to live with it. That’s the most important thing: having support. Whether it’s social, family, or medical, if you have that support, you can survive... – P7 (Woman, 47) |
